# Supplementary material for: Inflammation-Responsive Nanovalves of Polymer-Conjugated Dextran on a Hole Array of Silicon Substrate for Controlled Antibiotic Release
Source: Polymers (Basel). 2022 Sep 1;14(17):3611. doi: 10.3390/polym14173611 (PMC9459923; doi:10.3390/polym14173611)
Supplement: Supplementary file 1 [file polymers-14-03611-s001.zip › polymers-1821052-supplementary.pdf]

Article

Inflammation-Responsive Nanovalves of Polymer-Conjugated Dextran on a Hole Array of Silicon Substrate for Controlled Antibiotic Release

Ai-Wei Lee [1,2], Pao-Lung Chang [1], Shien-Kuei Liaw [3], Chien-Hsing Lu [4,5,*] and Jem-Kun Chen [2,*]

1 Department of Anatomy and Cell Biology, School of Medicine, College of Medicine, Taipei Medical University, Taipei 106, Taiwan
2 Department of Materials and Science Engineering, National Taiwan University of Science and Technology, Taipei 10607, Taiwan
3 Department of Electronic and Computer Engineering, National Taiwan University of Science and Technology, Taipei 10607, Taiwan
4 Department of Obstetrics and Gynecology, Taichung Veterans General Hospital, Taichung 40705, Taiwan
5 Ph.D. Program in Translational Medicine, Institute of Biomedical Sciences, Rong-Hsing Research Center for Translational Medicine, National Chung-Hsing University, Taichung 40227, Taiwan
* Correspondence: chlu@vghtc.gov.tw (C.-H.L.); jkchen@mail.ntust.edu.tw (J.-K.C.); Tel.: +886-2-27376523 (J.-K.C.); Fax: +886-2-27376544 (J.-K.C.)

(a)

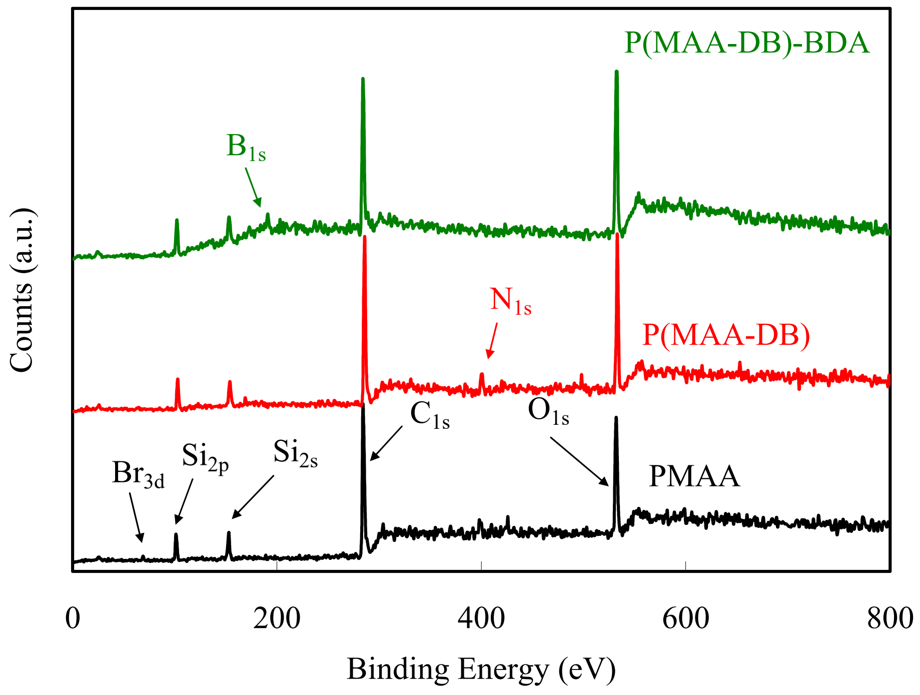

(b)

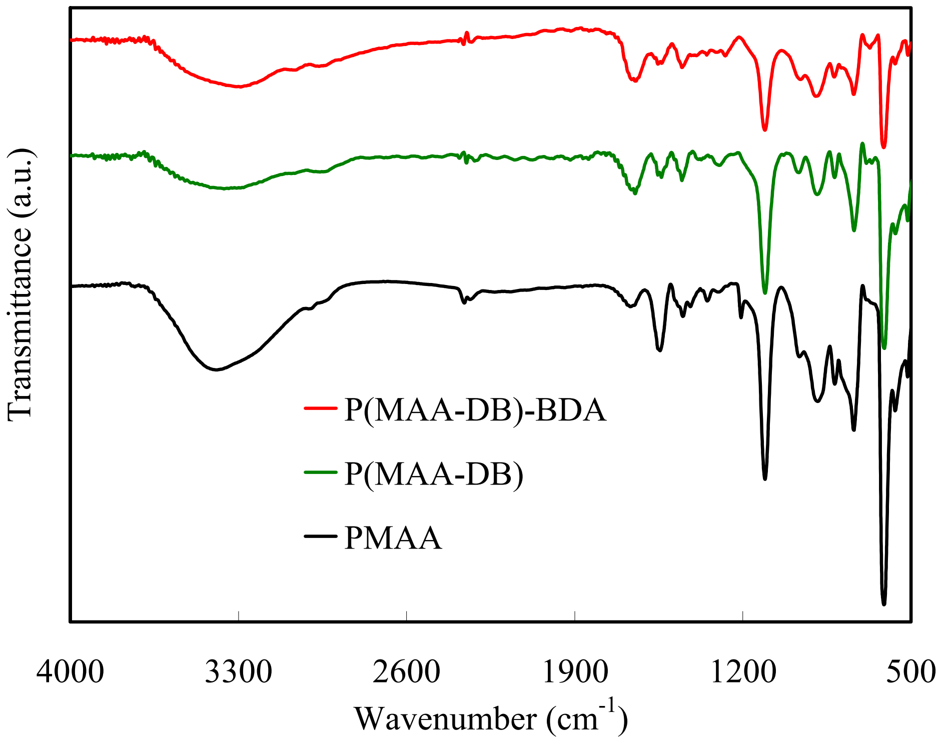

Figure S1. (**a**) XPS survey spectra and (**b**) FTIR spectra of 500H-PMAA, 500H-P(MAA-DB), and 500H-P(MAA-DB)-BDA.
